# Influence of lactide vs glycolide composition of poly (lactic-co-glycolic acid) polymers on encapsulation of hydrophobic molecules: molecular dynamics and formulation studies

**DOI:** 10.1007/s10856-021-06580-0

**Published:** 2021-09-30

**Authors:** Anurag Dobhal, Ashu Srivastav, Prajakta Dandekar, Ratnesh Jain

**Affiliations:** 1 Department of Chemical Engineering, Institute of Chemical Technology, Matunga, Mumbai, 400019 India; 2 Department of Pharmaceutical Sciences and Technology, Institute of Chemical Technology, Matunga, Mumbai, 400019 India

## Abstract

The work demonstrates the preparation of PLGA (PLGA 50:50, PLGA 75:25) nanoparticles, to encapsulate a hydrophobic molecule (coumarin-6), using the microreactor-based continuous process. The formulations were characterized using dynamic light scattering and transmission electron microscopy to determine their size, homogeneity, zeta potential, and surface morphology. The resulting nanoparticles were safe to the CHO cells (≈80% cell survival), at the concentration of ≤600 µg/mL and were successfully taken up by the cells, as demonstrated using confocal microscopy. Moreover, imaging flow cytometry confirmed that the nanoparticles were internalized in 73.96% of the cells. Furthermore, molecular dynamics simulation and docking studies were carried out to explore the effect of polymer chain length of PLGA and lactide vs glycolide (LA:GA) ratio on their compatibility with the coumarin-6 molecules and to study the coiling and flexibility of PLGA in the presence of coumarin-6 molecules. Flory–Huggins interaction parameter (*χ*) was calculated for polymer chains of varying lengths and LA:GA ratio, with respect to coumarin-6. *χ* parameter increased with increase in polymer chain length, which indicated superior interaction of coumarin-6 with the smaller chains. Amongst all the polymeric systems, PLGA55 exhibited the strongest interaction with coumarin-6, for all the chain lengths, possibly because of their homogeneous spatial arrangements and superior binding energy. PLGA27 showed better compatibility compared to PLGA72 and PGA, whereas PLA-based polymers exhibited the least compatibility. Analysis of the radius of gyration of the polymer chains in the polymer–coumarin-6 complexes, at the end of molecular dynamics run, exhibited that the polymer chains displayed varying coiling behavior and flexibility, depending upon the relative concentrations of the polymer and coumarin-6. Factors like intra-chain interactions, spatial arrangement, inter-chain binding energies, and polymer–coumarin-6 compatibility also influenced the coiling and flexibility of polymer chains.

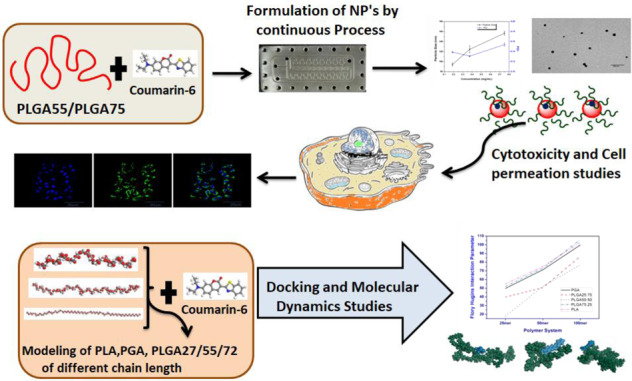

## Highlights

- Microreactor-based continuous process for the synthesis of PLGA nanoparticles.
- Miscibility of different PLGA polymers and coumarin-6 was evaluated computationally.
- Flory–Huggins interaction parameter (*χ*) was superior for shorter PLGA chains, irrespective of their lactide to glycolide ratio.
- Polymer chains containing lactide to glycolide ratio of 1:1 resulted in strongest interaction with coumarin-6 due to homogeneous spatial arrangements and superior binding energy.
- Intra-chain interactions, spatial arrangement, inter-chain binding energies, and polymer–coumarin-6 miscibility influence coiling and flexibility of polymer chains.

## Introduction

Particulate systems of biodegradable polymers have been extensively explored for controlled and targeted delivery of various drugs and biomolecules [1, 2]. Polymer-based dye-encapsulated fluorophores is one such system, extensively used to tag the drug delivery carriers and thus are extensively sought in pharmaceutical industry for imaging, bioanalytical, and targeting-based applications [3–5].

Amongst the FDA-approved biocompatible polymers, lactide (LA) and glycolide (GA) based poly (lactic-co-glycolic acid) (PLGA)-based copolymers have enormous potential as demonstrated by their extensive use in drug/protein/gene encapsulation [6–8], scaffold/stent preparation [9, 10], electronic, imaging devices [11, 12], etc. Composition of PLGA polymers can be varied by modulating the ratio of LA and GA repeat units and thus can alter its physicochemical and mechanical properties [13].

Formulation of PLGA-based delivery devices has been reported via various batch methods, including nanoprecipitation, emulsion-solvent evaporation, double emulsion-solvent evaporation, etc. [14, 15]. These methods employ high-speed homogenizers and are energy intensive. Moreover, these processes cannot be adequately controlled and hence result in variable particle formation, due to rapidity of mixing, nucleation, and particle growth [16]. In the present investigation we have prepared the nanoparticles by continuous process that enables the generation of reproducible and uniformly distributed nanoemulsions.

Since their introduction more than 4 decades ago, molecular dynamics (MD) simulations, in particular the classical MD and other computational methods, have been used to determine the solubility of pharmaceutically relevant molecules in various polymeric systems [17, 18]. MD and the other computational methods allow detailed understanding of not only the miscibility of drugs and excipients, but also help in investigating different factors and atomic level interactions that are important during the solubilization process [19, 20]. In pharmaceutical industry, MD has been extensively explored for free energy calculations, thermodynamics calculations, partitioning calculations, etc., which enable accurate prediction by integration of various algorithms and MD techniques [21–23]. MD simulations of PLGA-based polymers have been previously conducted to study their physicochemical properties, free energy calculations, and their interaction with various drug molecules, which cannot be deciphered via experimental methods [24, 25].

In this investigation PLGA–coumarin-6 nanoparticles were formulated using microreactor-based continuous process, which enabled formulation of size-controlled nanoparticles and resulted in batch-to-batch reproducibility. Furthermore, coumarin-6 was loaded into these nanoparticles using the same platform. Encapsulation of coumarin-6 was confirmed by investigating the uptake of labeled particles in mammalian cells, by confocal microscopy and imaging flow cytometry. Furthermore, the effect of LA vs GA ratio and molecular weight of PLGA on encapsulation of coumarin-6 was assessed via docking and atomistic MD simulations. The novelty of this work was in the development of a continuous platform for preparation of fluorescently labeled PLAG NPs and employment of detailed in silico computations to assess the stability of PLGA–coumarin-6 complexes. The research work conducted can be extrapolated to other hydrophobic molecules as well. These studies are of immense importance to understand the structural aspects of polymer for encapsulation of various actives during synthesis of newer nanoparticulate materials. This will expedite synthesis of reproducible polymeric nanoparticles for various applications. These investigations are relevant in several research areas such as design of novel materials, development of polymer-based drug delivery systems, computational material sciences, computational chemistry, etc. Throughout the manuscript, PLGA-based polymers have been referred as PLGAXY, where XY is 27 for the LA:GA of 25:75, 55 for LA:GA of 50:50, and 72 for LA:GA of 75:25.

## Materials and methods

### Materials

PLGA (Resomer^®^ RG 503H (Mw 24,000–38,000) and Resomer^®^ RG 752H (Mw 4000–15,000)) was obtained as a gift sample from Evonik India (Mumbai, India). Pluronics^®^ F68 (Poloxamer 188, poly (ethylene oxide) poly (propyleneoxide) block copolymer; average molecular weight 8400), paraformaldehyde, Dulbecco’s Modified Eagle Medium (DMEM), fetal bovine serum (FBS), Trypsin Phosphate Versene Glucose (TPVG), and Dulbecco’s phosphate-buffered saline (DPBS) were purchased from Hi Media Laboratories Pvt. Ltd (Mumbai, India). 3-(4, 5-Dimethyl-2-thiazolyl)-2, 5-diphenyltetrazolium bromide was purchased form (Sigma Chemical Co., St. Louis, MO, USA). MTT reagent (3-(4,5-dimethylthiazol-2-yl)-2, 5-diphenyltetrazolium) was purchased from Invitrogen (Mumbai, India). 4′, 6-Diamidino-2-phenylindole (DAPI) was purchased from ThermoFisher Scientific India Pvt Ltd (Mumbai, India). Acetone, dimethyl sulfoxide (DMSO), and glacial acetic acid were purchased from SD fine chemicals (Mumbai, India). HeLa cell line was procured from National Centre for Cell Sciences (Pune, India). Deionized, double-distilled water (Milli-Q Plus system, Millipore, MA, USA) was used throughout the study. All the chemicals used were of analytical grade.

### Formulation of PLGA and coumarin-6 nanoparticles

**Fig. 1:**
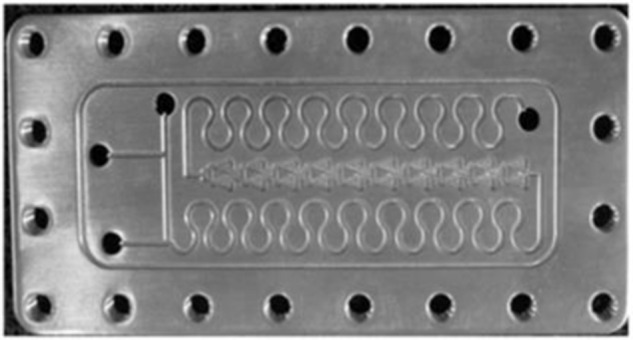
AMAR1 microreactor (https://amarequip.com/continuousflowmicroreactors/)

PLGA NPs were prepared using a microreactor, based on the principle of nanoprecipitation, as reported earlier [26]. Briefly, different amounts of PLGA were dissolved in acetone to prepare the organic phase of different concentrations, while the aqueous phase comprised of 0.04 mM solution of Pluronic F68. Two inlets of the microreactor were used to infuse the aqueous phase as against one inlet for infusing the organic phase. Flow rates of the aqueous and organic phases were selected such that the final volumes of the aqueous and organic phases, collected at the end of the residence time of 5 min, were 30 and 3 mL, respectively. The experiments were carried out at three concentrations of the polymers, viz. 0.18, 0.375, and 0.75 mg/mL. Coumarin-6-encapsulated nanoparticles were prepared by dissolving coumarin-6 in the organic phase, such that its concentration was 0.025% w/v. The microreactor used to formulate the nanoparticles was a commercial model (Amar Equipment Pt. Ltd, India), as depicted in Fig. 1. This reactor has an internal volume of 1 mL and comprises of a combination of a constant cross-sectional channel, having lamellar trajectory along the length, and a varying cross-sectional channel, having a straight trajectory. The design resulted in excellent mixing and offered a large area for heat transfer. The design details and details on the flow pattern of this micromixer have been previously published by Khalde et al. and Ranade et al. [27, 28].

### Particle size, size distribution, zeta-potential measurements

The average particle size (z-average), polydispersity index (PDI), and zeta potential of the nanoparticles were measured in triplicate, using dynamic light scattering (DLS) and laser doppler micro-electrophoresis (LDMe), respectively (Zetasizer Nano ZSP, Malvern, UK). DLS and LDMe were conducted at an angle of 173°, at 25 °C. Non-invasive back scatter technology was employed to yield maximum sensitivity, with the highest size and concentration range. Initial measurements were conducted on diluted NPs (10×). However, since dilution did not affect the mean particle size, further experiments were conducted on undiluted NPs.

### Transmission electron microscopy of the nanoparticles

Carbon/formvar-coated copper grids (200 #), used during the TEM studies, were purchased from Electron Microscopy Sciences PA, USA. TEM investigations were conducted using a ZEISS LIBRA Transmission Electron Microscope, operated at 120 keV. A drop of PMMA nanoparticles was taken on standard carbon formvar-coated copper grid (200 #), negatively stained with 2% magnesium uranyl acetate solution, for 30–60 s, and dried using 150 W lamp for 24 h. This procedure was conducted in a dust free zone, before recording the TEM. TEM analysis was performed on the PLGA55-coumarin-6 nanoparticles prepared with 0.375 mg/mL of the polymer.

### Purification and coumarin-6 encapsulation efficiency

Encapsulation efficiency (%EE) is the ratio of the amount of drug actually incorporated within the nanoparticles to the total amount added during formulation of nanoparticles. EE was calculated for PLGA55-coumarin-6 nanoparticles formulated using PLGA55 at the concentration of 0.375 mg/mL. The nanoparticle suspension (30 mL) was ultracentrifuged at 4 °C and 12,000 rpm for 40 min, using a CPR 24 PLUS REMI Tabletop cooling centrifuge (REMI, India) having the fixed angle rotor R-247 M. This was done to separate the un-encapsulated drug from the nanoparticles. The concentration-free coumarin-6 in the supernatant was further analyzed by HPLC. The measurements were performed in triplicate. HPLC analysis was conducted using Agilent 1290 infinity series HPLC, equipped with quaternary pump, thermostat, auto-sampler, and a diode array detector (DAD). Separation of the analytes was performed using an RP C18 column (reversed phase, Agilent TC-C18(2), 250 × 4.6 mm, pore size: 5 µm), wherein the mobile phase comprised of a mixture of acetonitrile and water (50:50 v/v), pumped at a flow rate of 1 mL/min. The column temperature was maintained at 25 °C with a column oven. The DAD was set at the wavelength (*λ*) of 227 nm for coumarin-6 detection. EE was calculated as per the formula stated in Eq. 1.

1 $${{{\rm{\% EE}}}} = \frac{{M_{{{{\rm{initial}}}}\,{{{\rm{drug}}}}} - M_{{{{\rm{free}}}}\,{{{\rm{drug}}}}}}}{{M_{{{{\rm{initial}}}}\,{{{\rm{drug}}}}}}}$$

where *M*_initial drug_ is the amount of drug added during nanoparticle preparation and *M*_free drug_ is the amount of the drug present in supernatant after centrifugation of the formulation.

### Determination of in vitro drug release kinetics

Drug release kinetics were determined for coumarin-6-loaded PMMA55 nanoparticles, prepared using the microreactor. Aliquots of nanoparticles (15 mL) were placed in a series of semi-permeable dialysis tubes (molecular mass cutoff 12,000 Da, Hi Media Laboratories Pvt. Ltd.), which in turn were immersed in 1-L PBS reservoir to mimic the infinite sink condition. Samples were collected from the dialysis tubes at predetermined time intervals and the amount of coumarin-6 remaining in the NPs at each release time point was measured by HPLC.

### Cell lines and culture media

CHO cells were cultured using DMEM supplemented with 10% FBS, in 37.5 cm^2^ flasks. The cells were cultivated by incubating for 24 h, at 37 °C, in an incubator (Galaxy^®^ 170 S; New Brunswick TM, Eppendorf AG, Germany) to provide an atmosphere composed of 95% air and 5% CO_2_. Before use, the cells were allowed to grow until confluence and were detached from flask using 1X TPVG solution containing 0.1% Trypsin, 0.02% EDTA, 0.05% glucose, and phenol red in DPBS. Subsequently, the cell density was determined using an automated cell counter (Countess II FL Automated Cell Counter). This cell counter operated on the basis of trypan blue staining, combined with an autofocus mechanism, and a sophisticated image analysis algorithm to obtain accurate cell and viability counts in 10 s. DPBS balanced salt solution was used for the handling and culturing of mammalian cells.

### Determining the cytotoxicity of the nanoparticles

The cellular cytotoxicity due to NPs was determined using MTT (3-[4, 5-dimethylthiazol-2-yl]-2, 5-diphenyltetrazolium bromide) assay [29]. The cytotoxicity of nanoparticles was performed on the PLGA55-coumarin-6 nanoparticles prepared with 0.375 mg/mL of the polymer. Briefly, CHO cells were seeded in 96-well plates, at the density of 10,000 cells per well. The cells were allowed to grow for 24 h, at 37 °C, in the presence of 5% CO_2_ and 95% relative humidity. NP samples, at the concentrations of 200, 400, 600, 800, and 1000 μg/mL, were incubated with the cells, after sterilizing the NPs by exposure to UV irradiations for 20 min, in a biosafety cabinet (ESCO Airstream Class II type Biological Safety Cabinet; ESCO Technologies, Hatboro, PA). Furthermore, the cell culture medium was replaced with 200 μL of NP samples and the cells were incubated for 24 and 72 h. At these time intervals, the medium containing NPs was removed and the cell monolayers were washed with DPBS (pH 7.4). Thereafter the cells were incubated for 4 h with 100 μL of the culture medium containing 0.5 mg/mL of MTT. Subsequently, the MTT solution was removed and 100 μL of DMSO was added to each well to dissolve the formazan crystals formed from MTT. The viability of cells treated with NP was determined by measuring the amount of formazan at 570 nm, with background correction at 630 nm. The amount of formazan was considered to be directly proportional to the number of living cells in the culture. All the samples were assayed in triplicate. Cell viability (%) of NPs was calculated according to Eq. 2.

2 $${{{\rm{Cell}}}}\,{{{\rm{viability}}}}\,\left( \% \right) = \frac{{ABs\,\left( {{{{\rm{Sample}}}}} \right)}}{{ABs\,\left( {{{{\rm{Control}}}}} \right)}} \ast 100$$

where *ABs*(Sample) is the absorbance recorded from the well treated with test sample and *ABs*(Control) is the absorbance measurement from the well without test sample, but treated with MTT.

### Cellular uptake of the nanoparticle via flow cytometry and confocal microscopy

#### Confocal microscopy

Approximately 10,000 CHO cells were seeded in a four-chambered cover slip, in complete medium, and allowed to grow till 70% confluence. The cells were then incubated with PLGA NPs, loaded with coumarin-6. Confocal microscopy was performed on the PLGA55-coumarin-6 nanoformulations prepared with the organic phase of 0.375 mg/mL concentration. After 4 h of incubation, the cells were washed twice with DPBS and fixed with 4% paraformaldehyde for 15 min, at 4 °C. After fixing, the cells were once again washed with DPBS. The nuclei of the cells were stained with DAPI (1 μg/mL) by incubating with the dye for 5 min. The cellular uptake of the NPs was visualized by a laser scanning confocal microscope (Leica Microsystem, Germany). Images were captured along the *z* axis to determine the intracellular localization of NPs.

#### Imaging flow cytometry

Imaging flow cytometry was performed with PLGA55-coumarin-6 nanoparticles prepared with 0.375 mg/mL of the polymer. CHO cells were seeded (1e^6^ per well) in 6-well plates in DMEM medium containing serum, for 24 h. Cells were treated with fluorescently labeled PLGA NPs for 24 h and washed with PBS, followed by trypsinization using 0.25% trypsin-EDTA (Gibco^®^, Life Technologies), at 37 °C, for 4 min. The trypsinized cells were suspended in 1 mL of DMEM containing 10% FBS and centrifuged at 1100 rpm for 4 min, at 4 °C. After aspiration of the medium, the cells were washed twice with 1 mL of PBS, at 1100 rpm, for 4 min, at 4 °C. After 24 h, the cells were harvested, fixed with 4% PFA, washed with PBS followed by staining with DAPI and CellMask stain. Data acquisition for internalization of the fluorescent NPs was conducted using Amnis imaging flow cytometer. Results were analyzed using FACS analysis software IDEAS^®^ (Image Data Exploration and Analysis Software) (Luminex Corporation, USA).

#### Modeling and preliminary geometry optimization

**Fig. 2:**
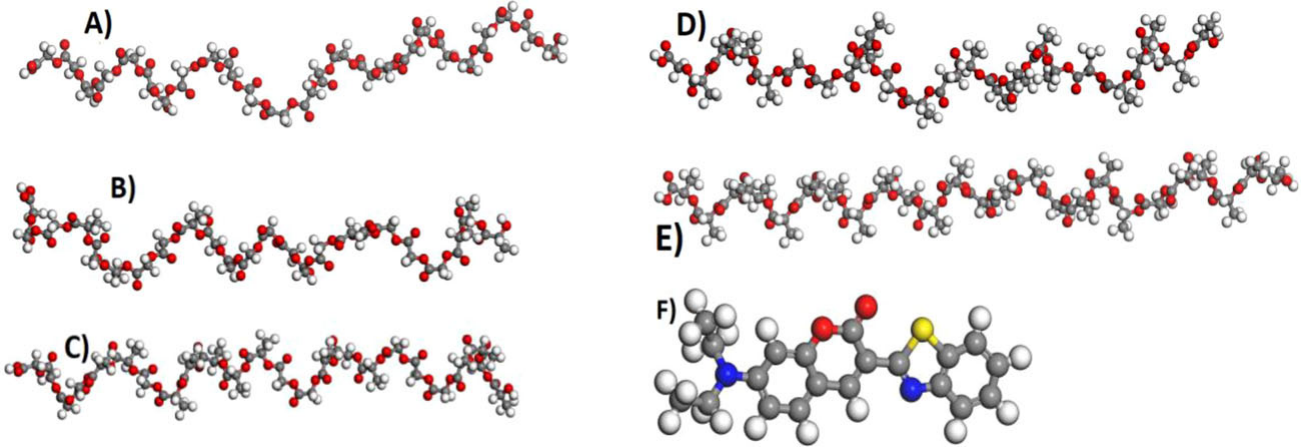
**A** 25mer PGA chain, **B** 25mer PLGA27 chain, **C** 25mer PLGA55 chain, **D** 25mer PLGA72 chain, **E** 25mer PLA chain, and **F** molecular structure of coumarin-6 (in all pictures, the chains LA and GA monomers were added randomly). All the structures presented here are geometry optimized. Coumarin-6 molecule exhibited energy of –37.58 kcal/mol after geometry optimization

All the polymer chains were modeled using polymer builder module, while the dye molecule (Fig. 2B) and repeat units for the polymers were modeled using the 3D atomistic platforms of Dassault Systèmes BIOVIA, Material Studio (MS), R2, San Diego: Dassault Systèmes [2017]. Preliminary geometry optimization of the chains and coumarin-6 was carried out using forcite module of MS. Conjugate gradient algorithm [30], with 500,000 steps, was used and convergence thresholds for maximum energy and maximum force changes were considered as 1.0e^−4^ Kcal/mol and 0.005 Kcal/mol/Å, respectively. Atomic charges were assigned by COMPASS force field [31, 32]. Periodic boundary conditions were not considered during the preliminary geometry optimization. The packing density of polymer cells was defined using the experimental bulk density of 1.50 g/cm^3^ for PGA [33], 1.44 g/cm^3^ for PLGA 25:75 [34], 1.34 g/cm^3^ for PLGA 50:50 [35], 1.30 g/cm^3^ for PLGA 75:25 [34], and 1.25 g/cm^3^ for PLA [35, 36], respectively (Table 1).

**Table 1:** Characteristics of various polymer chains considered for docking and MD calculations

Polymer chain	Monomers/length (Å) of the polymer chain after GO^a^	Potential energy (*E*_*T*_) of the chain after GO (Kcal/mol)	Mass (g/mol)
PGA	25/55.83	1.199	1468.91
	50/113.80	1.157	2919.81
	100/218.43	1.057	5821.61
PLGA27	25/52.74	1.312	1495.04
	50/118.22	1.236	3218.24
	100/232.74	1.227	6172.29
PLGA55	25/ 55.04	1.285	1579.20
	50/119.49	1.258	3400.59
	100/224.19	1.322	6522.96
PLGA72	25/52.74	1.477	1663.36
	50/118.21	1.327	3582.94
	100/244.64	1.307	6873.64
PLA	25/63.22	1.432	1819.59
	50/125.57	1.575	3621.16
	100/251.25	1.418	7224.31

Since various chain lengths were studied, total energy (*E*_*T*_) per atom has been represented

*GO* geometry optimization

^a^All the systems were converged after the geometry optimization

### Docking of different PLGA chains and coumarin-6 molecules

The miscibility of coumarin-6 with different PLGA chains was investigated by docking calculations, based on Flory–Huggins theory [37], as implemented in the blend’s module of MS. Different chain lengths of 25mer, 50mer, and 100mer and all five different compositions of PLGA-based polymers were considered for calculating the Flory–Huggins interaction parameter. The position of coumarin-6 molecule was kept fixed during the calculations and treated as the base, while the polymer chains were treated as screens that adopted various orientations around the base molecule. A total of 10,00,000 pair configurations were generated for each polymer chain-coumarin-6 pair during the calculation. From the resulting binding energies, the mixing energy (*E*_mix_) was calculated using Eq. 3 and subsequently the Flory–Huggins interaction parameter (*χ*) was determined using Eq. 4, to define the mutual interaction. Lower the value of *χ*, the higher the mutual compatibility (i.e., miscibility) of the pair. Mixing energy (*E*_mix_) represents the difference between free energies of the pure state and mixed state of both the components (base and screen). In equation 3, *Ebs* and *Esb* are the binding energies between one base and screen molecule each, while *Ebb* and *Ess* are the binding energies between two base and two screen molecules, in pure components. As the pair interactions *Ebb* and *Ess* are negative at the equilibrium state, the value of *E*_mix_ depends on the mutual ratio of absolute values of *Ebs*, *Esb*, *Ebb*, and *Ess*. This means that for a high miscibility, the value of *E*_mix_ will be negative.

3 $$E_{{{\rm{mix}}}} = \frac{1}{2}z\left( {Ebs + Esb - Ess - Ebb} \right)$$

4 $$\chi = \frac{{E_{{{\rm{mix}}}}}}{{RT}}$$

In Eq. 4, *R* is molar gas constant (Kg m^2^ K^–1^ S^–2^) and *T* is temperature (K). For the calculation of the co-ordination number (*Z* in Eq. 3), 10,000 cluster samples and 20 iterations per cluster were used. All the calculations were conducted at 298 K.

### Molecular dynamics and final geometry optimization for radius of gyration (*R*_*g*_) and for pair correlation function (*g*(r)) analysis

Initial models for MD, containing polymer chains and coumarin-6, were built under periodic boundary conditions [38], using amorphous cell module of MS. All the systems considered during MD simulations have been stated in Table 2.

**Table 2:** Details of the systems studied for the molecular dynamics simulations

Polymer chain	No. of polymer chains/no. of dye molecules	Weight % of coumarin-6	Weight % of PLGA
25mer PGA	1/1	19.26	80.73
50mer PGA	1/1	10.71	89.28
100mer PGA	1/1	5.67	94.32
25mer PLA	1/1	16.14	83.85
50mer PLA	1/1	8.82	91.17
100mer PLA	1/1	4.62	95.37
25mer PLGA 25:75	1/1	18.98	81.01
50mer PLGA 25:75	1/1	9.81	90.18
100mer PLGA 25:75	1/1	5.37	94.62
25mer PLGA 50:50	1/1	18.16	81.83
50mer PLGA 50:50	1/1	9.34	90.65
100mer PLGA 50:50	1/1	5.09	94.9
25mer PLGA 75:25	1/1	17.4	82.59
50mer PLGA 75:25	1/1	8.9	91.09
100mer PLGA 75:25	1/1	4.85	95.14
50mer PGA	3/1	3.84	96.15
50mer PGA	3/3	10.71	89.28
50mer PGA	3/6	19.35	80.64
50mer PGA	3/10	28.57	71.42
50mer PLA	3/1	3.12	96.87
50mer PLA	3/3	8.82	91.17
50mer PLA	3/6	16.21	83.78
50mer PLA	3/10	24.39	75.60
50mer PLGA 25:75	3/1	3.5	96.49
50mer PLGA 25:75	3/3	9.8	90.18
50mer PLGA 25:75	3/6	17.88	82.11
50mer PLGA 25:75	3/10	26.63	73.36
50mer PLGA 50:50	3/1	3.32	96.67
50mer PLGA 50:50	3/3	9.34	90.65
50mer PLGA 50:50	3/6	17.08	82.91
50mer PLGA 50:50	3/10	25.46	74.43
50mer PLGA 75:25	3/1	3.15	96.84
50mer PLGA 75:25	3/3	8.9	91.09
50mer PLGA 75:25	3/6	16.36	83.63
50mer PLGA 75:25	3/10	24.58	75.41

MD simulations were carried out using forcite module and all the charges were assigned using COMPASS force field. Initial models for MD calculations were generated using amorphous cell/construction module of MS, under periodic boundary conditions. NPT statistical ensemble was used with Nose thermostat [39], at *T* = 298 K and Berendsen borostat [40], at atmospheric pressure *p* = 101.325 kPa. Length of production run trajectory of 5 ns was found to be sufficient for the systems studied. For each polymer/dye structure, the last frame of trajectory was optimized after the dynamics run, at the same conditions as that of the preliminary geometry optimization (section “Materials”). Pressure was the same as that used for dynamics run and the unit cell in the periodic structure was treated without any constraints. Flexibility of polymer chains was fitted to parameters that affected the thermal and mechanical behavior. The effect of flexibility of different chains on the encapsulation of coumarin-6 was evaluated by determining the radius of gyration of the polymer chain in the PLGA–coumarin-6 complex at the beginning (*R*_*g*0_) of dynamic trajectory and at the end (*R*_*g*_), before the final geometry optimization.

The ratio *R*_*g*_*/R*_*g*0_ was used as a parameter to characterize the flexibility of the polymer chain. Higher the value of *R*_*g*_*/R*_*g*0_, greater was the difference between *R*_*g*_ and *R*_*g*0_, which indicated a higher flexibility of the chains. The radius of gyration of the polymer chain in the polymer-NBO complex was computed using Eq. 5.

5 $$R_g = \surd I/\surd M$$

where *t* is the moment of inertia and *M* is the total mass of all the atoms in the polymer–coumarin-6 complex.

Pair correlation functions or radial distribution function (*g*(r)) that gives the probability of finding a particle in a distance *r* from another particle was also calculated from MD simulation trajectories by keeping the cutoff distance of 20 Å and was checked at the interval of every 0.02 Å.

## Results and discussion

### Preparation and characterization of PLGA–coumarin-6 fluorophores

**Fig. 3:**
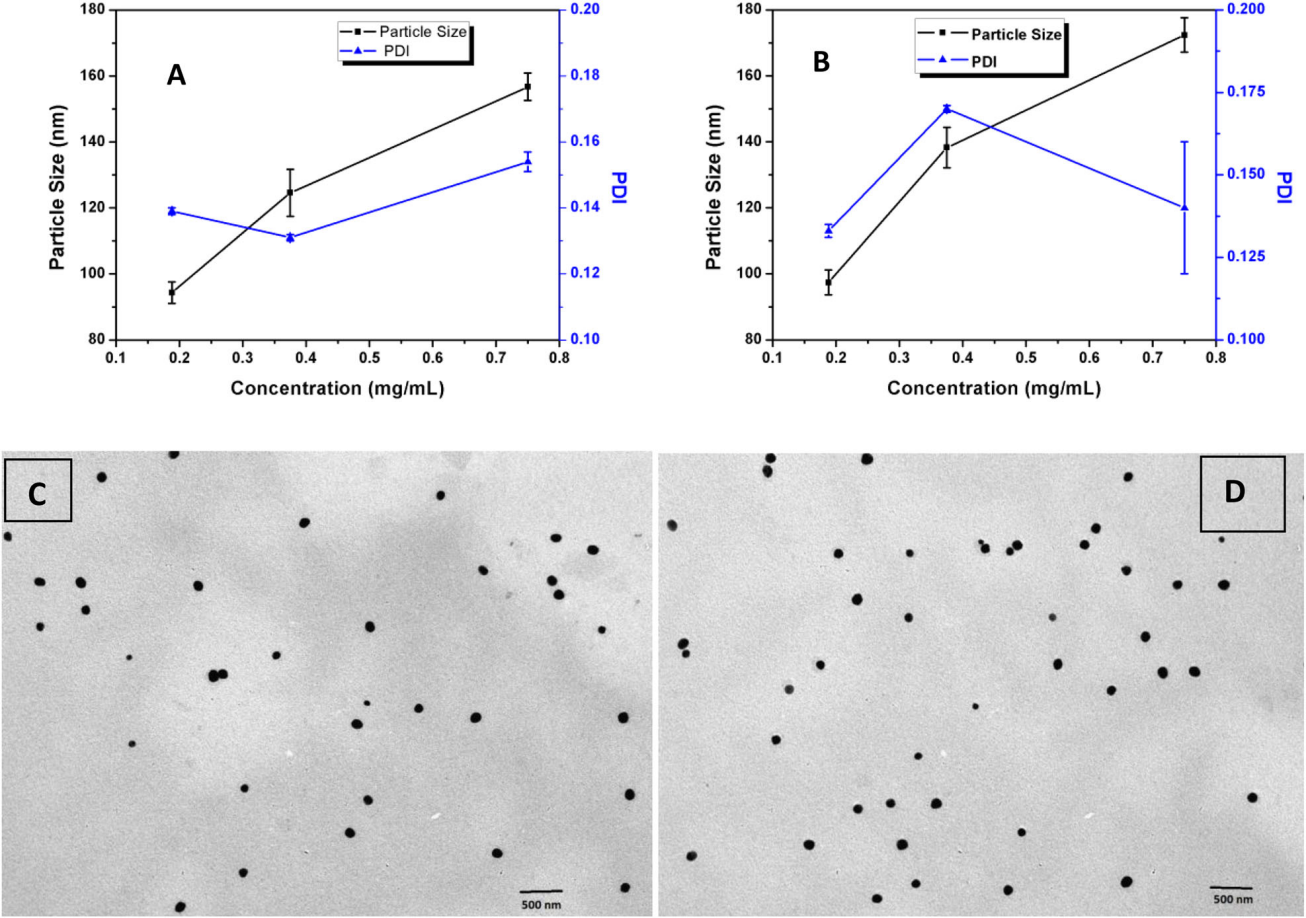
Size and PDI values. **A** PLGA55-coumarin-6, **B** PLGA72-coumarin-6 particles with varying polymer concentrations in the organic phase. **C**, **D** TEM images of PLGA55-coumarin-6 nanoparticles

Continuous platform for the formulation of PLGA NPs, using both the grades of polymers, resulted in monodisperse particulate suspensions, as suggested by the PDI values of the suspensions that were in the range of 0.131 ± 0.001 and 0.17 ± 0.001. The particle size of the nanoparticulate suspensions increased as the concentration of the polymer in the organic phase was increased (Fig. 3A, B and Supplementary Table S1).

Comparison of the particle size of NPs formulated using both the polymers, at different concentrations, illustrated that at lower concentration, the NPs formulated using both the polymers were almost equal in size. However, at higher concentrations, the NPs formulated using PLGA75 exhibited a slightly higher diameter. Increase in particle size with increasing concentration of the polymers was ascribed to the higher viscosity of the organic phase at higher polymer concentrations, which resisted precipitation of organic phase in the aqueous phase. At higher polymer concentrations, the organic phase droplets were more viscous and hence a higher surface tension may have existed between the aqueous and organic phases. This may have led to a lower diffusion of the polymer phase into the aqueous phase, resulting in larger solidified particles upon solvent evaporation [41, 42].

Increase in the size of NPs due to increase in polymer concentration in the organic phase has been earlier reported during batch-synthesis of nanoparticles. Similar results have also been reported by our group and other researchers working with microreactors for nanoparticle synthesis [26, 43]. Figure 3C, D depicts the TEM images of PLGA55-coumarin-6 nanoparticles prepared with 0.375 mg/mL of the polymer. The TEM images revealed a particle size of around 90 nm, whereas the DLS results showed that particles had an average size of 125 nm. This may be because the latter records the hydrodynamic mean diameter of the nanoparticles, in contrast to TEM where the drying step strips the hydration layer around the nanoparticles.

Further analyses, i.e., %EE cytotoxicity and cell permeation of coumarin-6 encapsulated NPs were conducted only with the nanoparticles formulated using PLGA55 having concentration of 0.375 mg/mL in organic phase, which exhibited an average size of 124.61 ± 7.1 nm. This formulation was selected considering the uniformity of the particles achieved as confirmed by the TEM analysis.

### Encapsulation efficiency (%EE) of coumarin-6-loaded nanoparticles

EE reveals percentage of the drug encapsulated in the polymeric particles, which is representative of particle and process efficiency. In our experiments coumarin-6-loaded PLGA55 nanoparticles were prepared in continuous mode, using a microreactor. The concentration of the coumarin-6 in the organic phase was 0.025% w/v. The drug to polymer ratio in the optimized nanoparticulate suspension was 1:15 w/w. The EE was observed as 55.3%, which confirmed that the microreactor technology could be successfully employed for preparation of molecule-loaded polymeric nanoparticles.

### In vitro drug release kinetics of the coumarin-6-loaded nanoparticles

**Fig. 4:**
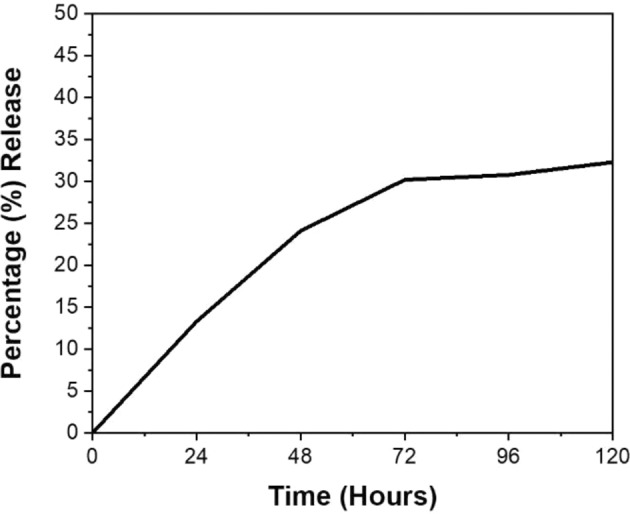
Release kinetics of coumarin-6 from PLGA55 nanoparticles

Experiments for studying the release kinetics revealed that after 24 h, 13.3% of coumarin-6 was released from the nanoparticles. After 48 h, 24.11% coumarin-6 was released, while after 72, 96, and 120 h, 30.2, 30.78, and 32.3% of the coumarin-6 was released from the PLGA55 nanoparticles, respectively. The results have been depicted in Fig. 4. This indicated that these particles could retain around 70% of coumarin-6 till the duration of 96 h and thus could be employed for the in vivo imaging studies.

### Cytotoxicity analysis of PLGA–coumarin-6 fluorophores

**Fig. 5:**
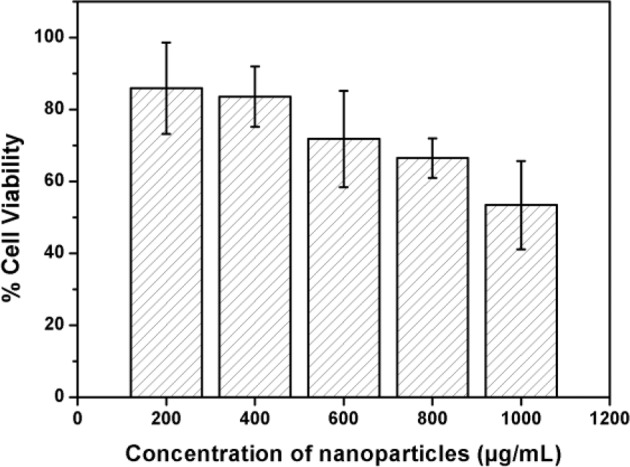
Cytotoxocity of coumarin-6-PLGA nanoparticles at different concentrations. Results have been presented as mean ± SD (*n* = 4)

Cytotoxicity analysis of the PLGA55-coumarin-6 nanoparticles prepared at the concentration of 0.375 mg/mL has been depicted in Fig. 5, which showed that higher concentrations of PLGA55-coumarin-6 nanoparticles lowered the cell survival rate as the highest cell survival of ≈90% was observed at the particle concentration of 200 µg/mL, which gradually reduced to ≈50% at 1000 µg/mL of NPs. These experiments confirmed the suitability of PLGA–coumarin-6 NPs for cellular evaluations at concentrations of ≈400 µg/mL.

### Cell permeation studies of PLGA–coumarin-6 fluorophores

The percentage of fluorescence-positive cells with internalized nanoparticles was acquired during the imaging flow cytometry experiments. The results indicated that coumarin-6-loaded PLGA NPs interacted with and adhered to the surface of CHO cells. Furthermore, the fluorescence-positive cells were analyzed for the percentage of cells showing an internal fluorescence for coumarin-6-loaded NPs. Here, cells having fluorescent NPs adsorbed on their surfaces outside the cells resulted in negative internalization values, whereas the cells that had internalized the NPs exhibited positive internalization scores. A total of 10,000 events were acquired for the entire sample population, wherein only 9800 cells were gated as single healthy cells for further analysis, while the remaining 1200 cells were either cell debris or doublet population.

**Fig. 6:**
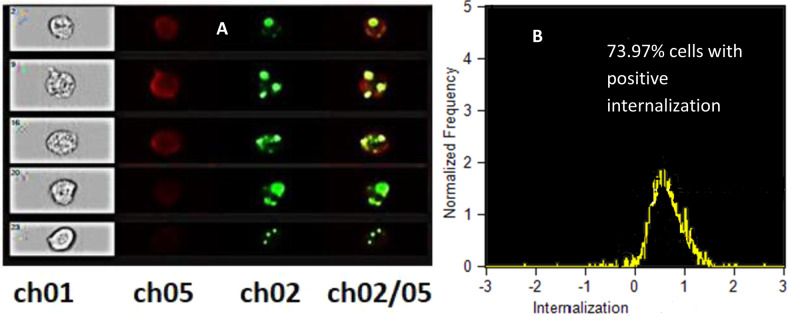
Uptake of coumarin-6-loaded PLGA NPs by CHO cells using imaging flow cytometry. Representative images captured by the Amnis Image StreamX Flow Cytometer of cells treated with D 73.97% cells with positive internalization score coumarin-6-loaded PLGA NPs for 4 h, at 37 °C. **A** First column (Ch01) shows bright-field (BF) images of the cells, second column (Ch05) shows images of fluorescence of CellMask for cell membrane, third column (Ch02) shows images of fluorescence of coumarin-6-loaded PLGA NPs, and fourth column (Ch02/05) shows the overlapping signals from columns 02 and 05. **B** Normalized frequency versus internalized cells for the internalization score (IS) calculated by Amnis IDEAS software. Scale bar in all the flow cytometry images is 20 µm

In Fig. 6A, the first column (represented as Ch01) shows bright-field images of the cells, the second column (represented as Ch05) depicts the fluorescence of CellMask for cell membrane, the third column (represented as Ch02) represents the fluorescence of coumarin-6-loaded PLGA NPs, and the fourth column indicates the overlapping signals of the columns 02 and 05. In this case, as shown in Fig. 6B, 73.97% of the cells exhibited a positive internalization score, as analyzed by the IFC analysis software IDEAS^®^ 6.0, affirming that most of the PLGA NPs were delivered inside the cells.

**Fig. 7:**
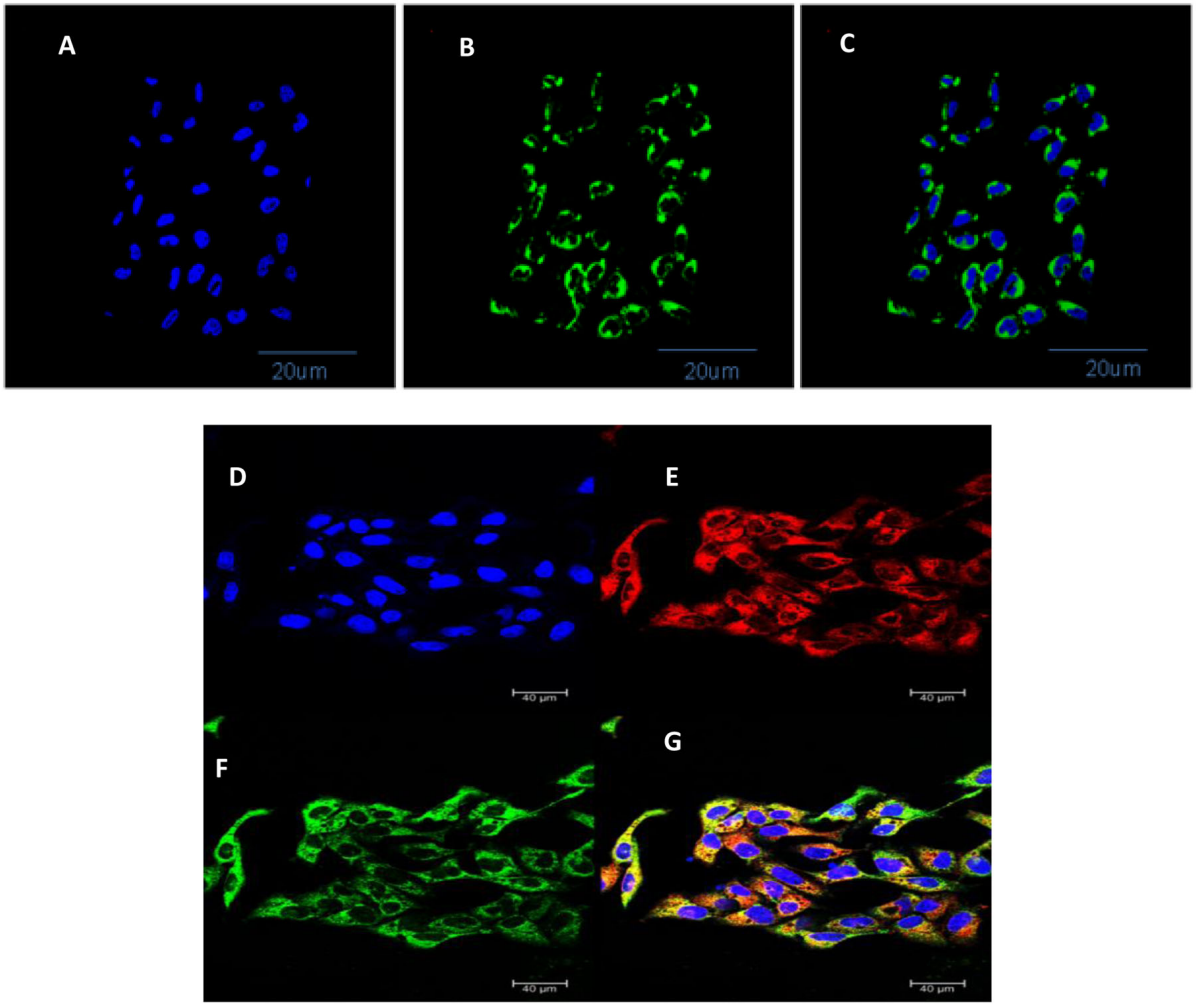
Results of the confocal microscopy images for cellular internalization of coumarin-6-loaded PLGA NPs. **A** is image of channel 1 that shows nucleus of the cells stained with DAPI (blue). **B** shows channel 2 showing the presence of coumarin-6-loaded PLGA NPs (Green) in the cellular cytoplasm. **C** shows channel 3, demonstrating an overlay of channels 1 and 2. **D** is the image of channel 1 that shows nucleus of the cells stained with DAPI (blue). **E** shows channel 2, which depicts staining of cell membrane with CellMask. **F** shows channel 3, showing the presence of coumarin-6-loaded PLGA NPs (green) inside the cellular cytoplasm. **G** shows channel 4, representing overlay of all the channels. Scale bar in all the confocal microscopy images is 40 µm

Furthermore, confocal microscopy was performed to confirm the intracellular localization of PLGA–coumarin-6 nanoparticles within the cytoplasm of the CHO cell lines (Fig. 7A–C). The images show evident presence of nanoparticles within cytoplasm. Also, the *z*-stack analysis of these images further confirmed the localization of these nanoparticles inside the cells and in between the plasma membrane and the nucleus (Fig. 7D–G).

### Results of docking calculations of PLGA-based polymers and coumarin-6 molecules

**Fig. 8:**
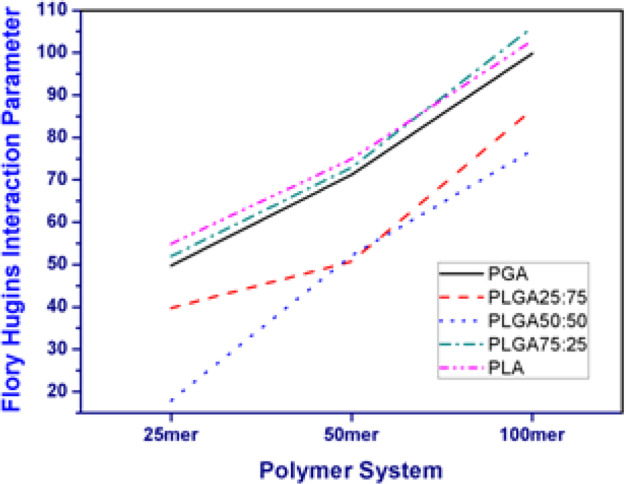
Comparison of Flory–Huggins Interaction parameter (*χ*) calculated for all the polymer–coumarin-6 complexes

**Fig. 9:**
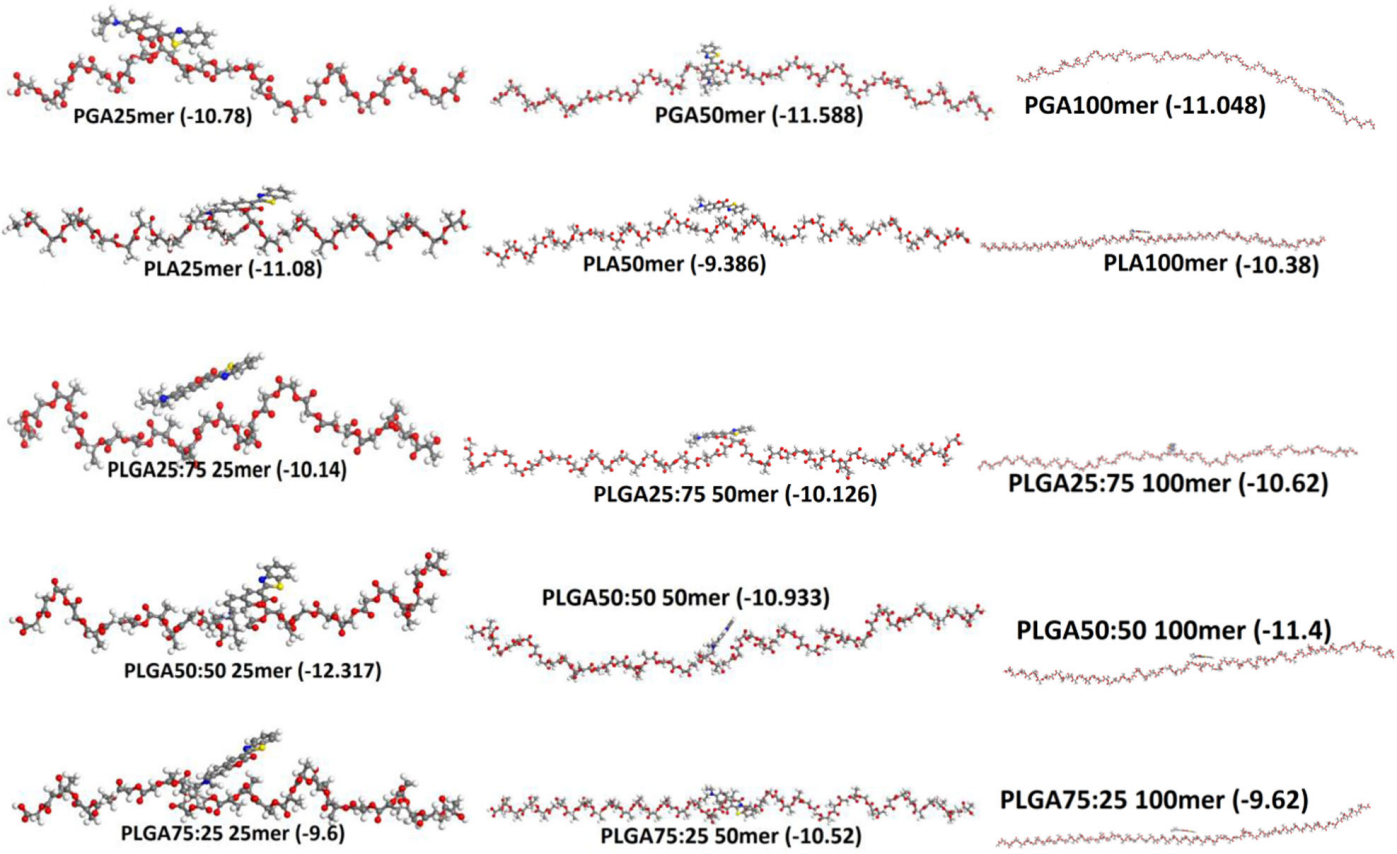
Lowest energy conformations of the polymer chains and coumarin-6 complexes obtained after docking calculations. Energies of the complexes have also been represented in graphical form in the figure. Exact values of the conformations have been stated in Supplementary Table S2

Docking calculations (Fig. 8) demonstrated that PLGA50:50 chains were the most appropriate to form complexes with coumarin-6, as per their miscibility data (Flory–Huggins interaction parameters). In case of the 25mer and 100mer chains, the Flory–Huggins interaction parameter for PLGA50:50 chains was the lowest among the values for all PLGA polymers. In case of 50mer chains, the interaction parameter for PLGA50:50 was equal to those of PLGA25:75 and was the lowest amongst all the other PLGA-based polymers. The interaction parameters for PLGA25:75 demonstrated superior miscibility as compared to those for PLGA75:25, for all the three chain lengths. The PGA chains displayed considerably improved miscibility as compared to the PLGA75:25 chains, for all the chain lengths, while the PLA chains demonstrated the lowest miscibility with coumarin-6 molecules, for 25mer and 50mer chain lengths. However, their miscibility was equivalent to that of the 100mer chains of PLGA75:25. The *χ* parameter of these polymer chains with coumarin-6 molecules was dependent on their mixing energy, which was in turn dependent on the binding energy of the polymer chains with themselves (*Ess*), the polymer chains with dye molecules, the dye molecules with polymer chains (*Ebs*, *Esb*), and of the dye molecules with themselves (*Ebb*), as stated in Eq. 3. The lowest energy conformations obtained after docking calculations are depicted in Fig. 9.

**Fig. 10:**
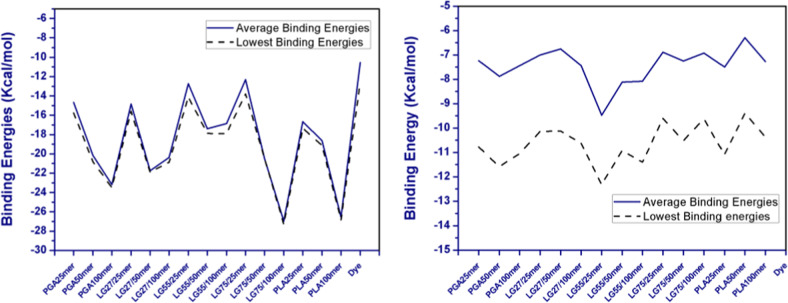
The average and lowest binding energies of the **A** different polymer chains with self **B** polymer chains with coumarin-6 molecules

The *χ* parameter of these polymer chains with coumarin-6 molecules was dependent on their mixing energy, which was in turn dependent on the binding energy of the polymer chains with themselves (*Ess*), the polymer chains with dye molecules, the dye molecules with polymer chains (*Ebs*, *Esb*), and of the dye molecules with themselves (*Ebb*), as stated in Eq. 3. Since all the binding energy values were negative (Fig. 10 and Supplementary Table S2), the *Ebb* and *Ess* increased the *χ* parameter, unlike *Esb* and *Ebs*. The binding energies of the polymer chains with themselves increased as the polymer chain length increased. Analysis of the binding energies of the polymer chains demonstrated highest values for the PLA–PLA chains, which were similar to the values obtained for PLGA75–PLGA75 chains. The lowest binding energies were observed for PLGA55 chains with themselves, while the binding energies of PLGA27–PLGA27 and PGA–PGA chains were comparable. The highest binding energies of PLA–PLA and PLGA75–PLGA75 chains were attributed to the strong attractive interactions among the methyl groups in these chains, along with the restricted rotations around the C-O bond [44]. Both these factors were also responsible for the higher values of the glass transition temperature of the PLA polymer [45]. Mobility of the PLGA55 chains was higher as compared to other PLGA-based polymers, as was observed from the flexibility data of the MD simulation (Table 3). The binding energy values of polymer chains with coumarin-6 molecules (Fig. 9 and Supplementary Table S2) demonstrated that in the polymer chain length range investigated that the binding energy values for all the polymer chains were between 6.294 land –9.480 Kcal/mol. As compared to other polymer chains, the binding energy was slightly higher for all the three 25mer, 50mer, and 100mer PLGA55 chains, i.e., –9.480, –8.118, and –8.078 Kcal/mol, respectively. Thus, the results demonstrated the suitability of PLGA55 for encapsulating coumarin-6 molecules as PLGA55 resulted in a more homogeneous network, providing sufficient functional groups and intramolecular regions for the dye molecules to mend within the polymer chains. In case of PLA chains, the intermolecular regions within the network of the polymer chains were densely occupied with methyl groups, while the PGA chains lacked the presence of sufficient functional groups to form non-bonded interactions with coumarin-6 molecules. Thus, both the binding energy factors, i.e, base–base binding energy and base–screen binding energy contributed toward the lower *χ* parameters values for PLGA55-coumarin-6 complexes. Overall, the results suggested that apart from the mixing energy of the polymer chain and coumarin-6, the spatial arrangement of the polymer chain also played a critical role in defining its miscibility with the coumarin-6 molecules. Thus, PLGA27 and PLGA72 can have different *χ* parameters, depending upon the placement of LA residues in the chain, as they are constituted randomly during modeling of the polymer chain.

**Table 3:** *R*_*g*_/*R*_*g*0_ of polymer chain in the presence of coumarin-6 molecule^a^

	PGA	PLGA27	PLGA55	PLGA72	PLA
25mer	1.08893	1.02868	0.97763	0.94325	1.03765
50mer	0.96188	1.01052	0.99768	0.97206	0.98093
100mer	1.00951	1.02351	0.97903	1.00031	1.03454
50mer system with 1 C molecule	0.962	1.005	0.97	0.979	1.032
	1.056	0.966	0.998	0.998	1.011
	0.955	0.993	0.991	1.007	1.025
50mer system with 3 C molecule	1.073	0.958	0.977	0.966	1.037
	1.071	0.971	0.989	1.013	1.014
	1.014	1.032	0.972	0.984	0.953
50mer system with 6 C molecule	1.03	1.004	1.041	0.927	1.034
	0.98	1.011	1.002	0.954	1.069
	1.02	1.001	0.978	1.019	1.017
50mer system with 10 C molecule	1.002	0.982	1.061	1.005	0.979
	1.002	0.969	0.888	0.984	1.001
	1.095	0.977	1.016	0.957	0.987

^a^Three values in each cell represent the *R*_*g*_/*R*_*g*0_of three different polymer chains in those systems

### Results of molecular dynamics simulations

#### Radius of gyration of the individual PMMA chains with single PLGA–coumarin-6 complexes and visual observations of the models

**Fig. 11:**
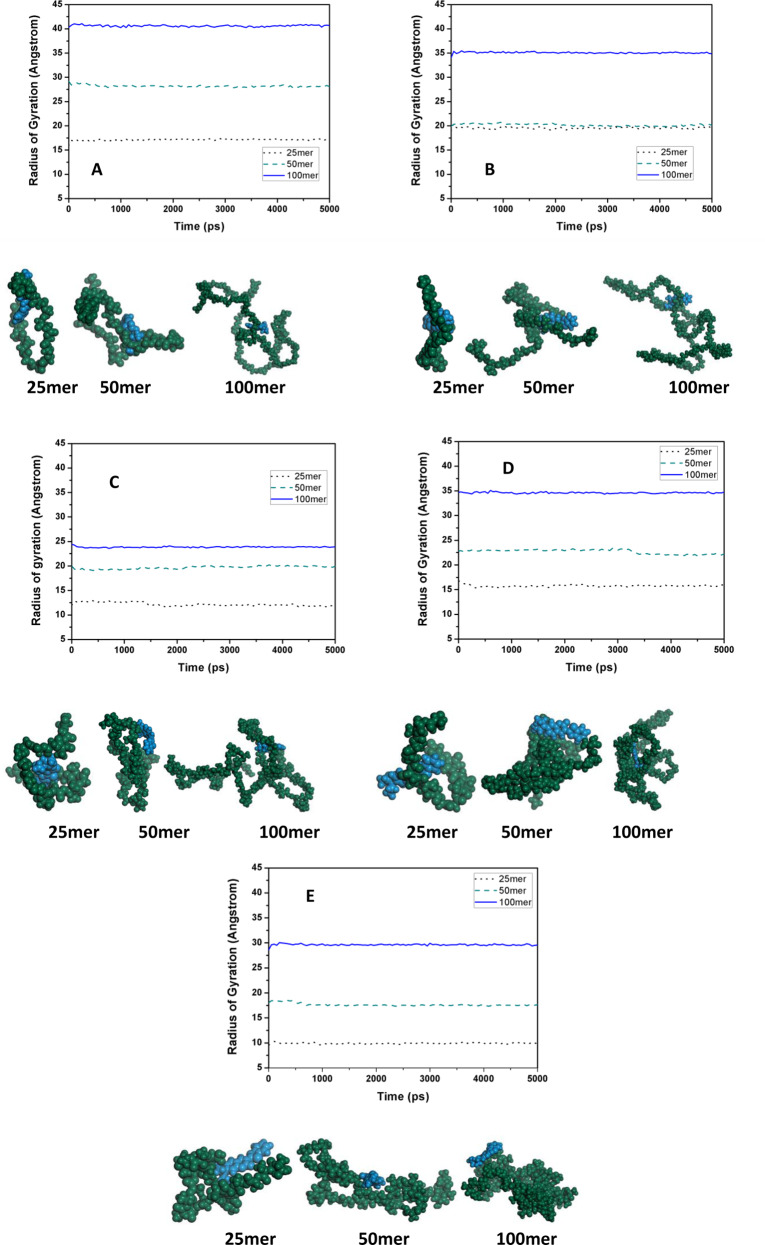
Change in radius of gyration of the polymer chains during the course of simulation time of 5 ns for **A** PGA, **B** PLGA27, **C** PLGA55, **D** PLGA72, and **E** PLA. Below each graph, the geometry optimized conformation of polymer–coumarin-6, obtained after 5 ns of molecular dynamics simulation has been shown

Radius of gyration (*R*_*g*_) is defined as the distance from the axis of rotation to a point where the total mass of the body (*M*) is supposed to be concentrated, so that the moment of inertia (*I*) about the axis remains the same [46]. It indicates the approximate size/radius of the object as *R*_*g*_ is directly proportional to *R* for all the physically defined shapes. In case of macromolecules, *R*_*g*_ is more meaningful, than the end-to-end distance, as it gives an estimate of size of the polymer coils [46–49]. Values of radius of gyration for the different polymer chains (Fig. 11) indicated that for all the polymer systems, the 100mer polymer chains had the highest radii of gyration, followed by the 50mer and 25mer chains, respectively. In case of PGA, the 100mer chain exhibited the highest *R*_*g*_ of 40.69 Å. PLGA55 exhibited the lowest *R*_*g*_ of 23.91 Å. The *R*_*g*_ for PLGA27 and PLGA72 was approximately 34 Å, while it was 29.57 Å for PLA. The pattern for the 50mer chains was as follows, *R*_*g*_ of 28.06 Å for PGA, *R*_*g*_ of 22.18 Å for PLGA72, *R*_*g*_ of 20.13 Å for PLGA27, *R*_*g*_ of 19.97 Å for PLGA55, and *R*_*g*_ of 17.67 Å for PLA. In case of the 25mer chains, the *R*_*g*_ was 17.31 Å for PGA, 19.53 Å for PLGA27, 11.69 Å for PLGA55, 15.91 Å for PLGA72, while the *R*_*g*_ was 9.95 Å for PLA.

Radius of gyration of any polymer depends upon the number of repeat units in the polymer chains; thus, larger chains have higher values of *R*_*g*_ [50, 51]. Thus, from the results it was inferred that for 100mer and 50mer chains, the PGA chains formed the largest coiled structure, followed by PLGA27 and PLGA72. On the other hand, PLGA55 and PLA formed the smallest coils. The PLA chains are highly hydrophobic owing to the abundance of methyl group in their chains, which resulted in these chains forming smaller coils due to the attractive interactions between the methyl groups present within the chains [44]. These attractive interactions among the methyl groups may have led to intra-chain attractions, thus producing smaller coiled structures. On the other hand, PLGA55 chains have regular-ordered spatial arrangement in their chains, besides having molecular groves in their structures, unlike the PLA chains. PLA chains have methyl groups in all their monomers, while PLGA55 has methyl groups only in the alternate monomers. The molecular groves offer improved spatial arrangements to inhabit functional groups in the chains, producing smaller coiled structures as lesser space is occupied by the chains. The largest coiled structure of the individual PGA chains was attributed to the fact that these chains completely lacked any intra-chain attractive forces due to the absence of methyl groups. Thus, PLGA27 and PLGA72 formed coiled structures having *R*_*g*_ values higher than those of PLA and PLGA and lower than those of PGA.

##### Pair correlation function analysis of the PLGA–coumarin-6 complexes

Radial distribution function of the PMMA–NBO complexes exhibits (Supplementary Fig. S3) that the complexes of NBO with all five types of PLGA-based polymers can be formed when distance between both the entities is around >0.95 Å, but below this distance the complexes will not be formed because of strong repulsive forces. Furthermore, ≈3–8 times likelihood of the presence of complexes can be observed when the distance between the entities lies between 1.04 and 1.13 Å and ≈2–4 times likelihood when the distance between them is around 2 Å. Higher probabilities were observed in the case of PLA and PLGA75 polymers that can be ascribed to the presence of increased number of hydrophobic methyl groups in their chains resulting in higher like interactions with hydrophobic coumarin-6 molecules.

#### Radius of gyration of polymer chains in PLGA–coumarin-6 complexes where more than one PMMA and coumarin-6 are present in the system

**Fig. 12:**
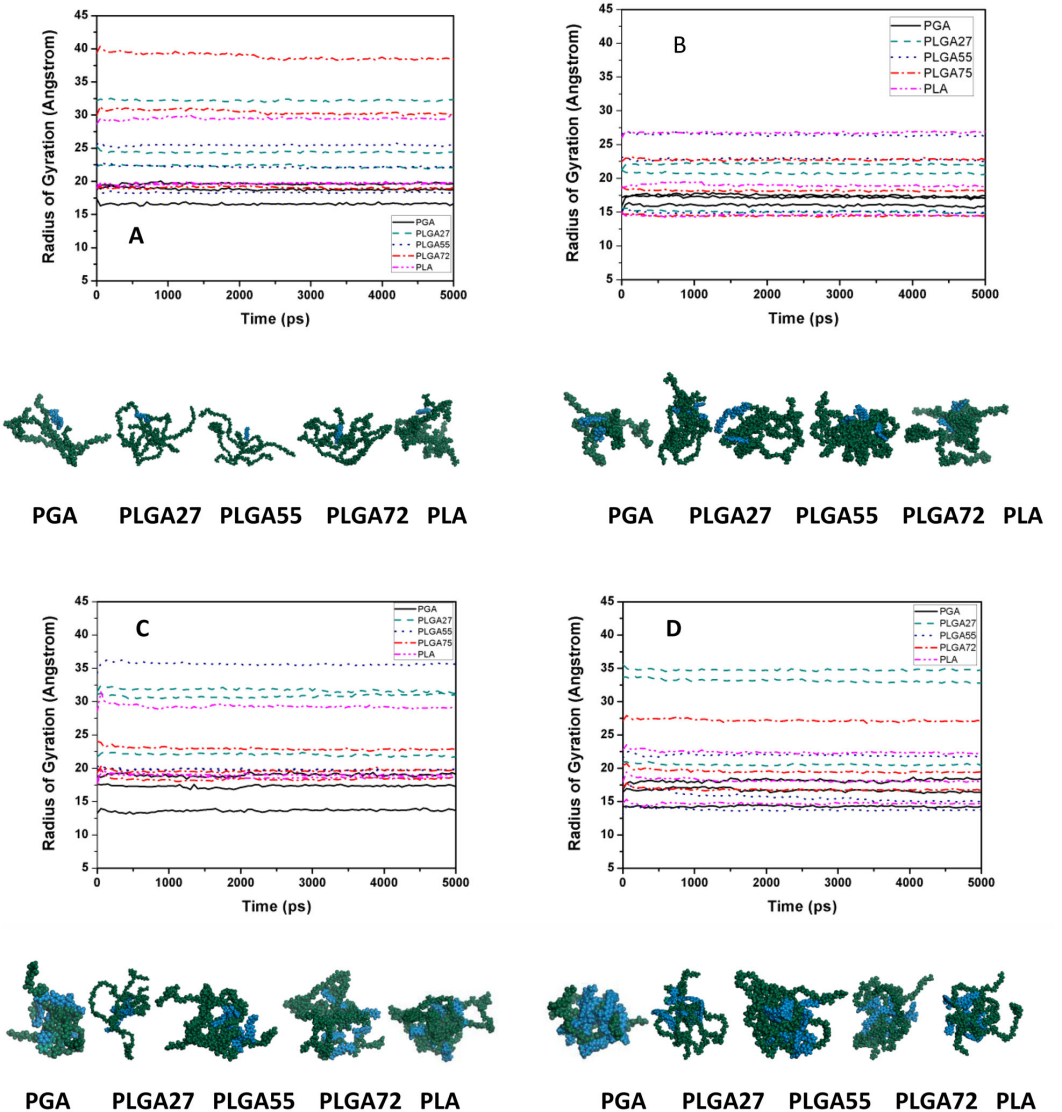
Change in radius of gyration of the polymer chains during the course of simulation time of 5 ns for **A** three 50mer chains of each polymer type with one coumarin-6 molecule, **B** three 50mer chains of each polymer type with three coumarin-6 molecule, **C** three 50mer chains of each polymer type with sixcoumarin-6 molecule, and **D** three 50mer chains of each polymer type with ten coumarin-6 molecule. Below each graph, the geometry optimized conformation of polymer–coumarin-6 obtained after molecular dynamics simulation of 5 ns has been shown

The behavior of the PLGA chains in the presence of coumarin-6 molecules was further understood by analyzing the *R*_*g*_s of the polymer chains, when simulated in groups, and in the presence of more than one dye molecules (Fig. 12). This was conducted by simulating the polymer chains in systems consisting of three 50mer polymer chains with varying number of dye molecules, i.e., 1, 3, 6, and 10. Analysis of the results of all the different systems demonstrated that *R*_*g*_s of different PLGA-based polymers did not follow any particular trend but different chains of a particular system exhibited an erratic relation with any particular chain of different polymeric systems. Another observation was that unlike the results of simulation of a single chain with a single drug molecule, the PGA chains did not form large coiled structures but had smaller *R*_*g*_s for all the chains, in all the systems studied, as compared to the *R*_*g*_ value of the 50mer PGA chains, when simulated singleton. This behavior of PGA chains is attributed to their highly crystalline and dense solid structures [52]. From the binding energy data, it was seen that the PGA chains possessed inter-chain binding energies, as compared to the other polymers under consideration (Fig. 9 and Supplementary Table S2). Observing the behavior of the 50mer PLA chains, simulations showed that as compared to its *R*_*g*_ value of 17.67 Å in single chain system, in three chain system with differing coumarin-6 molecules, the PLA chains exhibited *R*_*g*_ values ranging from 14.325 to 29.02 Å. However, further increasing the number of coumarin-6 molecules introduced randomness in the systems, resulting in differentially coiled PLA structures.

Similar to PLA, the other copolymers also lacked any particular pattern and had a wider distribution for all the polymers. Like in case of PLA chains, PLGA polymers of different compositions resulted in homogenous particles as the polymer concentration was increased. The main factor for this observation was that these polymers had similar binding energies. It was thus concluded that formulation of PLGA NPs had lower dependence on varying compositions of LA and GA residues in the polymers, but particles of desired sizes could be achieved by varying the concentrations of the polymers or by using polymers with varying molecular masses. However, composition of LA and GA residues may play a critical role during encapsulation and release of various active agents from NPs.

From the flexibility data (Table 3) of the polymer chains it was concluded that the PLGA55 chains possessed the highest flexibility as compared to other polymer systems, which was ascribed to their homogenous spatial arrangement along with their amorphous solid structure. The other polymer systems exhibited fewer flexible chains as compared to the PLGA55 chains.

From the visual observations of the geometry optimized conformations obtained after MD simulations for 5 ns, it was concluded that in case of the systems containing more than one dye molecule and one polymer chains (Fig. 12), the polymer–polymer chains preferred to form coiled structures, wherein most of the coumarin-6 molecules were found clustered together, forming non-bonded interactions with the polymer structures. This observation was made on the basis of the higher binding energies of the polymer–polymer chains, compared to the polymer–coumarin-6 complexes. This observation also suggested that the polymer chains preferred to self-assemble amongst themselves instead of forming dye-encapsulated particulate systems. Thus, while formulating NPs, external energy has to be provided to formulate polymer–coumarin-6 complexes.

## Conclusion

Experiments were carried out to formulate PLGA-encapsulated coumarin-6 nanoparticles using a microreactor-based continuous platform, based on the principle of nanoprecipitation. Two different polymers, differing in the ratio of LA and GA residues (LA:GA), i.e., 50:50 and 75:25, were employed for the preparation of the particles. Uptake of the NPS was conducted via in vitro experiments in CHO cell line. Encapsulation was confirmed by confocal microscopy and quantified using imaging flow cytometry. Toxicity of the particles using MTT assay demonstrated >80% survival of CHO cells, when the particle concentration was ≤600 µg/mL.

Effect of molecular weight (polymer chain length) and ratio of LA and GA residues in PLGA polymers was studied with respect to their ability to encapsulate coumarin-6 molecules. These were studied by performing docking and MD (5 ns) calculations. Polymers having five ratios, i.e., PLGA (100:0), PLGA (75:25), PLGA (50:50), PGA (0:100), and three chain lengths, i.e., 25mer, 50mer, and 100mer were studied for computational calculations. Docking calculations were based on calculation of Flory–Huggins interaction parameter, which confirmed superior compatibility of PLGA polymers having LA and GA in equal proportion. PLGA27 had higher compatibility than PLGA72 and PGA, whereas PLA chains exhibited the least compatibility with the dye molecules. Flory–Huggins interaction parameter increased upon increasing the polymer chain length, confirming the superior compatibility of the smaller chains. Radius of gyration of the polymer chains, over stimulation time of 5 ns, and flexibility analysis of the polymer chains exhibited varying characteristics of the different polymer chains. It was observed that characteristics of any individual polymer chains were influenced by the concentration of the polymer and the coumarin-6 molecules in the system. Moreover, increasing the concentration of coumarin-6, for a particular concentration of the polymer, produced randomness in the systems. At higher concentrations of the dye molecules, it became difficult to differentiate the structural properties of the polymer chains based on their LA vs GA ratio. Both the formulation and MD indicated that the size of nanoparticulates systems had a lower dependence on the ratio of LA and GA residues but were more affected by the concentration of the polymer. However, the encapsulation of coumarin-6 molecules is dependent on the ratio of LA and GA residues in PLGA polymers of different compositions.
